# Occurrence of mucosa-affecting diseases of the upper airways in middle ear cholesteatoma patients: a nationwide case–control study

**DOI:** 10.1007/s00405-024-08567-3

**Published:** 2024-03-22

**Authors:** Agnes Modée Borgström, Hanna Mogensen, Cecilia Engmér Berglin, Johan Knutsson, Åsa Bonnard

**Affiliations:** 1 Department of Clinical Sciences, Intervention and Technology, Karolinska Institutet, Stockholm, Sweden; 2 Medical Unit of ENT, Hearing and Balance, Karolinska University Hospital, Stockholm, Sweden; 3 Unit of Epidemiology, Institute of Environmental Medicine, Karolinska Institutet, Stockholm, Sweden; 4 Department of Otolaryngology, Västerås Hospital, Västerås, Sweden; 5 Faculty of Medicine and Health, Örebro University, Örebro, Sweden; 6 Region Västmanland, Centre for Clinical Research, Uppsala University, Västmanland Hospital, Västerås, Sweden

**Keywords:** Cholesteatoma, Rhinitis, Sinusitis, Adenoidectomy, Tonsillectomy, Nasal polyposis

## Abstract

**Purpose:**

Exploring a possible link between upper airway inflammation and the development of cholesteatoma by studying the association between mucosa-affecting diseases of the upper airways and cholesteatoma surgery.

**Methods:**

This is a nationwide case–control study of 10,618 patients who underwent surgery for cholesteatoma in Sweden between 1987 and 2018. The cases were identified in the National Patient Register and 21,235 controls matched by age, sex and place of residency were included from national population registers. Odds ratios (OR) and corresponding 95% confidence intervals were used to assess the association between six types of mucosa-affecting diseases of the upper airways and cholesteatoma surgery.

**Results:**

Chronic rhinitis, chronic sinusitis and nasal polyposis were more common in cholesteatoma patients than in controls (OR 1.5 to 2.5) as were both adenoid and tonsil surgery (OR > 4) where the strongest association was seen for adenoid surgery. No association was seen between allergic rhinitis and cholesteatoma.

**Conclusion:**

This study supports an association between mucosa-affecting diseases of the upper airways and cholesteatoma. Future studies should aim to investigate the mechanisms connecting mucosa-affecting diseases of the upper airways and cholesteatoma formation regarding genetic, anatomical, inflammatory and mucosa properties.

**Supplementary Information:**

The online version contains supplementary material available at 10.1007/s00405-024-08567-3.

## Introduction

The mucosa lining the tympanic cavity is continuous with the Eustachian tube, the nasopharynx, and the mastoid [1]. An association between chronic rhinosinusitis (CRS) and chronic otitis media (COM), a chronic inflammation of the middle ear, has been reported [2]. It can potentially be explained by the anatomical proximity through the Eustachian tube or by immunologic similarities of the mucosa [2, 3]. Eustachian tube dysfunction has also been shown to be associated with allergic rhinitis [4].

Cholesteatoma of the middle ear is a mass consisting of keratinizing squamous epithelium growing in the tympanic cavity and/or the mastoid [5]. The annual incidence of cholesteatoma ranges from 9 to 15 per 100 000 people, with a male preponderance [6]. Serious complications such as deafness, facial palsy, and intracranial infections occur and surgical treatment is, therefore, recommended [7]. The pathophysiology of cholesteatoma is relatively unknown and many biomolecular hypotheses have been suggested [8]. One theory is that cholesteatoma arises due to Eustachian tube dysfunction [7]. Another theory is that it may be the result of a reaction of the eardrum with an inflammatory dysregulation including immune cells and cytokines in the middle ear, that can be triggered by infection [9].

An association between CRS and cholesteatoma has previously been reported [3, 10]. One of the studies also showed an association between allergic rhinitis and cholesteatoma [10]. Another study showed positive associations between both CRS and nasal polyposis with cholesteatoma but a negative association between allergic rhinitis and cholesteatoma, partially contradicting the other studies [11]. To the best of our knowledge there is only one previous study investigating the association between allergy and cholesteatoma in a European setting [12]. Causes of adenoid and tonsil hypertrophy are insufficiently known, but it has been shown that recurrent infections, allergies, gastric reflux, and passive smoking are risk factors [13]. It has also been shown that adenoid hypertrophy may contribute to the cause of CRS [13].

The anatomically connected mucosa, the observed associations between CRS and chronic otitis media, as well as the association between allergies and Eustachian tube dysfunction, has prompted the hypothesis that there is an association between mucosa-affecting diseases of the upper airways and the occurrence of cholesteatoma. The inconclusive findings of previous studies underscore the importance of the present investigation. This nationwide register study aimed to explore the association between mucosa-affecting diseases of the upper airways and cholesteatoma. This was done by studying chronic rhinosinusitis, nasal polyposis, allergy, adenoid hypertrophy, and tonsillar hypertrophy in individuals with cholesteatoma compared to matched controls. Additionally, the present study seeked to analyze the potential effects of age, sex, and family history cholesteatoma on this association.

## Methods

### Study design

This was a nationwide case–control study including all patients who underwent surgical treatment for cholesteatoma in Sweden between 1987 and 2018. The cases were retrieved from the National Patient Register, a nationwide register of discharge diagnoses and treatments with national coverage from inpatient care facilities since 1987. For diagnoses and treatments received at specialized outpatient care facilities, the register has national coverage from 2001. Diagnoses from general outpatients care facilities were not reported in the register during the study period [14].

The cases were identified using diagnostic codes, according to the International Classification of Diseases (ICD 9 and ICD 10), in combination with surgical codes according to the Swedish Classification of Medical Procedures and the 6th edition of Swedish Surgical Classification for cholesteatoma surgery registered at the same visit. Diagnostic codes are listed in Supplement 1. Two controls per case, matched for age, sex, and place of residency at the date of cholesteatoma surgery (hereinafter referred to as the index date), were obtained from the Total Population Register [15].

### Exposure

Having a mucosa-affecting disease of the upper airways was defined as having one or more hospital visits for one of the following diagnoses: allergic rhinitis, chronic rhinitis, chronic sinusitis and/or nasal polyposis during any time between 1987 and 2018. For children < 15 years of age, having undergone adenoid, tonsillar or adenotonsillar surgery was also defined as having a mucosa-affecting disease of the upper airways. Both primary and secondary diagnoses were included and identified from the National Patient Register.

### Other covariates

Demographic characteristics such as age, sex, and years registered as a Swedish resident were obtained from the Total Population Register. Family history of cholesteatoma was defined as having one or more first-degree relative who underwent cholesteatoma surgery between 1987 and 2018. Relatives of the cases and controls were identified in the Multigeneration Register. The patients with no known first-degree relative (*n* = 1308, 4.1%) were excluded from the subgroup analysis of family history to enhance the accuracy of the analyses.

### Statistical analysis

Differences in categorical variables between cholesteatoma patients and controls, including family history of cholesteatoma and the presence of mucosa-affecting diseases of the upper airways, were analyzed using a chi-square test. For the comparison of the number of years registered as living in Sweden, the median was chosen due to a skewed distribution. For a comparison of the medians, the independent samples median test was used. Differences in the number of comorbidity diagnoses between cases and controls were analyzed using an independent samples *t* test. The association between mucosa-affecting diseases of the upper airways and cholesteatoma was investigated by estimating odds ratios (OR) and corresponding 95% confidence intervals (CI) using conditional logistic regression. A *P *value of < 0.05 was considered statistically significant. Stratified analyses by age, sex, family history of cholesteatoma, and index date before or after the year of 2000 were performed to examine the association between mucosa-affecting diseases of the upper airways and cholesteatoma in these subgroups. A sensitivity analysis was conducted excluding upper airway diagnoses received in the period 1 year before and 1 year after the index date. This analysis was performed to examine whether the variation in the number of diagnoses could be attributed to cases receiving medical attention for their cholesteatoma.

Data from the national registers were received in April 2022 and data analysis was performed from March to May 2023. All data analysis was performed using IBM SPSS Statistics for Windows version 25 (IBM) and SAS statistical software, version 9.4 (SAS Institute Inc).

## Results

A total of 10,618 patients were identified as surgically treated for cholesteatoma in Sweden between 1987 and 2018, and 21,235 matched controls were additionally included in the study. For one cholesteatoma case, there was only one matched control, resulting in one less person than expected in the control group. Of the participants in the study, 59.4% were male and 77.3% were over 15 years of age at the index date. The median number of years registered as living in Sweden was 32.0 years in both cases and controls (Table 1).

**Table 1:** Descriptive characteristics of patients surgically treated for cholesteatoma in Sweden between 1987 and 2018

Characteristics	Cases *N* (%)	Controls *N* (%)	*P *value
Total, number	10 618	21 235	NA
Age at index year, mean (SD)	35.6 (21.5)	35.6 (21.5)	NA
Age < 15 years at index year	2416 (22.7)	4832 (22.7)	NA
Male	6302 (59.4)	12 603 (59.4)	NA
Index year 1987–2000	5122 (48.0)	10 244 (48.0)	NA
Index year 2001–2018	5496 (52.0)	10 991 (52.0)	NA
Years registered as Swedish resident, median	32.0	32.0	NS
Mucosa-affecting diseases of the upper airways			
Having any mucosa-affecting disease	647 (6.1)	982 (4.6)	< 0.001
Three or more mucosa-affecting diseases	213 (2.0)	325 (1.5)	NS
Number of mucosa-affecting diseases, mean (SD)	3.5 (5.6)	3.5 (6.7)	NS
More than one kind of mucosa-affecting disease	84 (0.8)	96 (0.5)	< 0.001
Family history			
At least one relative identified	10 133 (95.4)	20 412 (96.1)	0.004
At least one relative with cholesteatoma	227 (2.2)	118 (0.6)	< 0.001

*NA* not applicable, *NS* not statistically significant

Mucosa-affecting diseases of the upper airways were more common in cholesteatoma patients compared to controls, as was having mucosa-affecting airway diagnoses of more than one kind. The mean number of mucosa-affecting diseases of the upper airways did not differ between the groups (Table 1).

There was an association between chronic rhinitis, chronic sinusitis and nasal polyposis and cholesteatoma. All three diagnoses were statistically significantly more common in patients treated for cholesteatoma compared to matched controls. Similar associations between mucosa-affecting diseases of the upper airways and cholesteatoma as in the main analyses were seen in individuals without a family history of cholesteatoma. However, the association could not be investigated among individuals with a family history of cholesteatoma. This was due to the small number of individuals with both a family history of cholesteatoma and a mucosa-affecting disease of the upper airways (Table 2).

**Table 2:** The association between upper airway inflammation and cholesteatoma, ORs and 95% CIs, total and stratified analyses

	Allergic rhinitis				Chronic rhinitis				Chronic sinusitis				Nasal polyposis			
	Cases (%)	Controls (%)	OR (95% CI)	*P *value	Cases (%)	Controls (%)	OR (95% CI)	*P*-value	Cases (%)	Controls (%)	OR (95% CI)	*P *value	Cases (%)	Controls (%)	OR (95% CI)	*P *value
Total	319 (3.0)	591 (2.8)	1.0 (0.9–1.2)	NS	175 (1.6)	181 (0.9)	1.9 (1.6–2.4)	< 0.001	118 (1.1)	155 (0.7)	1.5 (1.2–1.9)	< 0.001	127 (1.2)	163 (0.8)	1.6 (1.2–2.0)	< 0.001
Age < 15y	119 (1.1)	255 (1.2)	0.9 (0.7–1.2)	NS	20 (0.2)	34 (0.2)	1.2 (0.7–2.0)	NS^c^	14 (0.1)	11 (0.1)	2.5 (1.2–5.6)	0.016 ^c^	19 (0.2)	12 (0.1)	2.5 (1.3–5.0)	0.005
Age > 15y	200 (1.9)	336 (1.6)	1.2 (1.0–1.4)	NS	155 (1.5)	147 (0.7)	2.2 (1.7–2.7)	< 0.001	104 (1.0)	144 (0.7)	1.5 (1.1–1.9)	0.004	108 (1.0)	148 (0.7)	1.5 (1.1–1.8)	0.003
Family history^a,b^	8 (0.1)	< 5 (–)	–	–	< 5 (–)	< 5 (–)	–	–	< 5 (–)	< 5 (–)	–	–	< 5 (–)	< 5 (–)	–	–
No family history^a^	305 (2.9)	581 (2.7)	1.1 (0.9–1.3)	NS	165 (1.6)	179 (0.8)	1.9 (1.6–2.4)	< 0.001	111 (1.0)	152 (0.7)	1.5 (1.2–1.9)	0.001	124 (1.2)	158 (0.7)	1.6 (1.3–2.0)	< 0.001
Men	185 (1.7)	352 (1.7)	1.1 (0.9–1.3)	NS	88 (0.8)	105 (0.5)	1.7 (1.3–2.3)	< 0.001	68 (0.6)	82 (0.4)	1.7 (1.2–2.3)	0.002	89 (0.8)	122 (0.6)	1.5 (1.1–1.9)	0.006
Women	134 (1.3)	239 (1.1)	1.1 (0.9–1.4)	NS	87 (0.8)	76 (0.4)	2.3 (1.7–3.29)	< 0.001	50 (0.5)	73 (0.3)	1.4 (1.0–2.0)	NS ^c^	38 (0.4)	41 (0.2)	1.9 (1.2–3.0)	0.005
Index year 1987–2000	107 (1.0)	199 (0.9)	1.1 (0.8–1.4)	NS	73 (0.7)	78 (0.4)	1.9 (1.4–2.6)	< 0.001	55 (0.5)	77 (0.4)	1.4 (1.0–2.0)	0.043 ^c^	59 (0.6)	79 (0.4)	1.5 (1.1–2.1)	0.019
Index year 2001–2018	212 (2.0)	392 (1.8)	1.1 (0.9–1.3)	NS	102 (1.0)	103 (0.5)	2.0 (1.5–2.7)	< 0.001	63 (0.6)	78 (0.4)	1.6 (1.2–2.3)	0.004	68 (0.6)	84 (0.4)	1.6 (1.2–2.3)	0.003

The number and % of cases and controls refers to exposed individuals

*NS* not statistically significant

^a^Analysis only on patients with at least one known relative

^b^Not enough cases with family history for cholesteatoma to run analysis

^c^Not statistically significant if period 1 year before/after surgery is excluded

In a sensitivity analysis excluding diagnoses received in the period 1 year before and 1 year after the index date, no statistically significant association between chronic sinusitis and cholesteatoma was seen for patients with the index year 1987–2000, nor in patients < 15 years of age. The results of all other analyses remained statistically significant and the effect estimates remained similar (Table 2, Supplement 2).

Adenoid and tonsil surgery were statistically significantly more common in cholesteatoma patients compared to in controls. Adenoid surgery had the strongest association with an OR of > 4 in all subgroups. The association was seen in all the subgroups, with a *P* < 0.001 in all adenoid and tonsil surgery, as well as in adenoid surgery and tonsil surgery separately (Table 3).

**Table 3:** The association between adenoid and tonsil surgery and cholesteatoma in patients < 15 years of age, ORs and 95% CIs, total and stratified analyses

	All adenoid and tonsil surgery			Adenoid surgery			Tonsil surgery		
	Cases (%)	Controls (%)	OR (95% CI)	Cases (%)	Controls (%)	OR (95% CI)	Cases (%)	Controls (%)	OR (95% CI)
Total	463 (19.2)	408 (8.4)	2.7 (2.3–3.1)	256 (10.6)	139 (2.9)	4.4 (3.5–5.5)	266 (11.0)	310 (6.4)	1.8 (1.5–2.2)
Family history^a,b^	18	< 5	–	7	< 5	–	12	< 5	–
No family history^a^	442	404	2.6 (2.2–3.0)	247	137	4.5 (3.5–5.7)	252	308	1.8 (1.5–2.1)
Men	299 (12.4)	233 (4.8)	3.0 (2.4–3.6)	162 (6.7)	88 (1.8)	4.3 (3.2–5.7)	168 (7.0)	166 (3.4)	2.1 (1.7–2.7)
Women	164 (6.7)	175 (3.6)	2.2 (1.7–2.8)	94 (3.9)	51 (1.1)	4.6 (3.2–6.8)	98 (4.1)	144 (3.0)	1.4 (1.1–1.9)
1987–2000	180 (7.5)	167 (3.5)	2.4 (1.9–3.1)	63 (2.6)	27 (0.6)	5.6 (3.4–9.3)	128 (5.3)	151 (3.0)	1.8 (1.4–2.3)
2001–2018	283 (11.7)	241 (5.0)	2.8 (2.3–3.4)	193 (8.0)	112 (2.3)	4.1 (3.2–5.2)	138 (5.7)	310 (6.4)	1.9 (1.5–2.4)

The number and % of cases and controls refers to exposed individuals

^a^Analysis only on patients with at least one known relative

^b^Not enough cases with familial cholesteatoma to run analysis

## Discussion

This nationwide register study of cholesteatoma surgeries over a 30-year period showed a 1.5 to 2.5 times higher prevalence of chronic rhinitis, chronic sinusitis, and nasal polyposis in cholesteatoma patients compared to controls. An even stronger association was observed for adenoid and/or tonsil surgery where choelsteatoma patients operated before 15 years of age were more than four times more likely than controls to have undergone adenoid surgery.

Previous studies have reported conflicting results regarding the association of different types of mucosa-affecting diseases of the upper airways and cholesteatoma. Two studies have found a statistically significant association between CRS and cholesteatoma, with cholesteatoma being twice as common in patients with CRS [3, 10]. These findings align with the results in the present study. The factors linking CRS and cholesteatoma have been hypothesized to be obstruction of the Eustachian tube and similar biomolecular or cellular dysregulation [3]. Another study showed a significant association between CRS and COM, however no association between cholesteatoma and CRS was shown in contradiction to the present study [2].

Adenoid surgery has previously been shown to be associated with cholesteatoma, where the rate of cholesteatoma dropped following adenoid surgery in a population-based retrospective study [16]. However, in another study the association was not statistically significant [17]. Adenoid hypertrophy, the reason for adenoid surgery, is associated with recurrent infections and biofilm in the adenoid which has been shown to be more common if the patient also suffers from CRS [13]. Recurrent infections may also lead to otitis media with effusion (OME) which in turn may lead to a retraction of the ear drum and further possibly to cholesteatoma [18, 19]. However, caution is needed when interpreting the results since adenoid surgery sometimes is performed as treatment for eardrum retraction to prevent the formation of a cholesteatoma [16]. Ear drum retraction is not a common indication for tonsillectomy [20]. Therefore, the association between tonsillectomy and cholesteatoma is possibly related to factors in the mucous epithelium rather than to Eustachian tube obstruction or eardrum retraction. The immunological response in the mucosa seems to affect both the nose, the paranasal sinuses, the epipharynx, and the middle ear. This leads to a hypothesis that recurrent infections or biofilm may be the link between the mucosa-affecting diseases of the upper airways and cholesteatoma.

CRS is a heterogenous disease where different inflammatory pathways are involved within the subgroups with and without nasal polyposis, and there has been extensive work on characterizing the immune response in CRS [21]. In the present study, CRS was divided into chronic rhinitis and chronic sinusitis separately, and the term nasal polyposis did not include rhinosinusitis. This was due to the use of ICD diagnostic codes in the National Patient Register. The results of the present study showed that both CRS with and without nasal polyposis were associated with cholesteatoma. This is supported by a cross-sectional study where cholesteatoma was associated with nasal polyposis and postnasal drip, a sign of CRS [11]. Since CRS is a heterogenous disease, there may be variants of the disease that have a stronger correlation with cholesteatoma, something that was not possible to investigate in the present study. Previous studies of nasal polyps have shown that healthy tissue adjacent to the polyps share characteristics with the polyps on a molecular level [22]. In cholesteatoma adjacent tissue dysregulation of a pathway of cell growth and inflammation has been shown [23]. Perhaps there are changes in cholesteatoma adjacent tissue caused by mucosa-affecting diseases of the upper airways.

Allergy differs from the other mucosa-affecting upper airway diagnoses in terms of its non-infectious and seasonal and mixed seasonal-perennial nature [24], which could explain the lack of association in the present study. Previous studies show conflicting results regarding the association between allergic rhinitis and cholesteatoma [11, 12, 25, 26]. Allergies are triggered by the exposure to environmental allergens, resulting in an IgE-mediated response that activates various cytokines and chemokines [27]. This indicate that IgE may not be the main pathway associated with cholesteatoma development. Supporting this is the lack of efficacy of antihistamines as treatment for OME [18]. However, the present study did not permit evaluation of the subgroups of CRS where some include elevated levels of IgE [28]. Therefore, inference on the association between IgE-mediated inflammation and cholesteatoma could not be made in the present study. Future studies of the subgroups of CRS are warranted to better understand the underlying mechanisms of the association with cholesteatoma.

## Strengths and limitations

The use of data from the National Patient Register with national coverage of diagnoses provided objectively reported data for both cases and population-based controls. More cholesteatoma patients received upper airway diagnoses from different groups compared to controls. This could indicate that receiving medical attention for cholesteatoma leads to the identification of additional diagnoses. However, there was no statistically significant difference in the number of upper airway diagnoses between cases and controls. Furthermore, the sensitivity analysis where we excluded diagnoses received one year before or after the cholesteatoma surgery to account for increased surveillance of cholesteatoma patients, yield similar findings as the main analysis. This supports the association between mucosa-affecting diseases of the upper airways and cholesteatoma.

While the study provided valuable insights into the association between mucosa-affecting diseases of the upper airways and cholesteatoma, the findings may not be generalizable to other populations or healthcare systems. Another limitation is that changes in diagnostic criteria over time might have affected the results. There could also be a risk of faulty coding in both diagnostic and surgical codes. However, the register has a high sensitivity regarding surgical procedures [14]. In the present study only one surgical code for tonsillotomy was used. A more unspecific surgical code can be used but was not included in the present study which may have affected the results. However, this surgical code was omitted in both cases and controls and it has been decreasing in use since 2010 [29]. Furthermore, the design of the present study did not permit inference on the causality between mucosa-affecting diseases of the upper airways and cholesteatoma.

## Conclusions

In this nationwide case–control study of cholesteatoma surgeries over a 30-year period a higher occurrence of chronic rhinitis, chronic sinusitis, and nasal polyposis was seen in cholesteatoma patients compared to controls. An even stronger association was observed for adenoid and/or tonsil surgery where an the strongest association was seen for adenoid surgery in individuals < 15 years of age. Further research is warranted to better understand the mechanisms of the association between mucosa-affecting diseases of the upper airways and cholesteatoma in regard to genetic, inflammatory and mucosal properties.

### Supplementary Information

Below is the link to the electronic supplementary material.

Supplementary file1 (DOCX 18 KB)

Supplementary file2 (DOCX 20 KB)

## Data Availability

These data are available for research purposes through applications to each register holder after ethical review and secrecy assessment. Data is available from Statistics Sweden (https://www.scb.se/vara-tjanster/bestalla-mikrodata/), the Swedish National Board of Health and Welfare (https://bestalladata.socialstyrelsen.se/data-for-forskning/) after ethical approval.
