# Supplementary material for: Occurrence of mucosa-affecting diseases of the upper airways in middle ear cholesteatoma patients: a nationwide case–control study
Source: Eur Arch Otorhinolaryngol. 2024 Mar 22;281(8):4081–7. doi: 10.1007/s00405-024-08567-3 (PMC11266238; doi:10.1007/s00405-024-08567-3)
Supplement: Supplementary file 1 — Supplementary file1 (DOCX 18 KB) [file 405_2024_8567_MOESM1_ESM.docx]

# Supplement 1

## Definition of cholesteatoma

Diagnostic codes according to ICD 9: 385.3 or 383.3

Diagnostic codes according to ICD 10: H71.9, H95.0

Surgical codes according to the Swedish Classification of Medical Procedures, KVÅ:

DCA30, DCB00, DCD00, DCD10, DCW99, DDA10, DDD05/10/ 20, DEA10, DEB00/10/20/25/30/40, DED00.

Surgical codes according to the 6^th^ edition of Swedish Surgical Classification:

2042, 2052, 2056, 2059, 2070-78, 2080-88.

## Definition of mucosa-affecting diseases of the upper airways

| **Supplementary table 1.**  Upper airway diagnoses by group. | | |
| --- | --- | --- |
|  | **ICD 9** | **ICD 10** |
| Allergic rhinitis | 4770, 4778, 4779 | J30.1, J30.2, J30.3, J30.4 |
| Chronic rhinitis | 4720, 4722 | J31.0, J31.1 |
| Chronic sinusitis | 473x | J32x |
| Nasal polyposis | 4710, 4711, 4719 | J33.0, J33.1, J33.9 |

## Definitition of adenoid and tonsil surgery

Surgical codes for adenoid and/or tonsil surgery according to the Swedish Classification of Medical Procedures, KVÅ:

All adenoid and tonsil surgery: EMB10, EMB15, EMB20 and EMB30

Adenoid surgery: EMB30

Tonsil surgery: EMB10 and EMB15

EMB99 was not included in the study.
