# Supplementary material for: Occurrence of mucosa-affecting diseases of the upper airways in middle ear cholesteatoma patients: a nationwide case–control study
Source: Eur Arch Otorhinolaryngol. 2024 Mar 22;281(8):4081–7. doi: 10.1007/s00405-024-08567-3 (PMC11266238; doi:10.1007/s00405-024-08567-3)
Supplement: Supplementary file 2 — Supplementary file2 (DOCX 20 KB) [file 405_2024_8567_MOESM2_ESM.docx]

# Supplement 2

Sensitivity analysis where diagnoses of mucosa affecting diseases of the upper airways recieved within one year before and after the index date where excluded to analyse the effect of increased medical attention in cholesteaomapatients. This sensitivity analysis did not yield any statisticaly significant association between chronic sinusitis in children <15 years of age and for chronic sinusitis in patients with the index year between 1987 and 2000 and cholesteatoma. In the other analyses the significance remained.

| **Supplementary table 2.**  The association between upper airway inflammation and cholesteatoma when diagnoses received more than one year before or after index date are excluded, ORs and 95% CIs, total and stratified analyses. | | | | | | | | | | | | | | | | |
| --- | --- | --- | --- | --- | --- | --- | --- | --- | --- | --- | --- | --- | --- | --- | --- | --- |
|  | **Allergic rhinitis** | | | | **Chronic rhinitis** | | | | **Chronis sinusitis** | | | | **Nasal polyposis** | | | |
|  | **Cases**  **(%)** | **Controls**  **(%)** | **OR**  **(95% CI )** | **P-value** | **Cases**  **(%)** | **Controls**  **(%)** | **OR**  **(95% CI )** | **P-value** | **Cases**  **(%)** | **Controls**  **(%)** | **OR**  **(95% CI )** | **P-value** | **Cases**  **(%)** | **Controls**  **(%)** | **OR**  **(95% CI )** | **P-value** |
| Total | 296 | 563 | 1.1 (0.9-1.2) | NS | 162 | 174 | 1.9 (1.5-2.3) | <0.001 | 108 | 149 | 1.5 (1.1-1.9) | 0.003 | 116 | 159 | 1.5 (1.2-1.9) | 0.002 |
| Age <15y | 114 | 242 | 0.9 (0.7-1-2) | NS | 18 | 32 | 1.1 (0.6-2.0) | NS | 12 | 11 | 2.2 (1.0-4.9) | NS | 17 | 15 | 2.3 (1.1-4.5) | 0.021 |
| Age>15y | 182 | 321 | 1.1 (0.9-1.4) | NS | 144 | 142 | 2.1 (1.6-2.6) | <0.001 | 96 | 138 | 1.4 (1.1-1.8) | 0.012 | 99 | 144 | 1.4 (1.1-1.8) | 0.014 |
| Family history ^a, b^ | 7 | <5 | - | - | <5 | <5 | - | - | <5 | <5 | - | - | <5 | <5 | - | - |
| No family history ^a^ | 284 | 555 | 1.1 (0.9-1.2) | NS | 155 | 173 | 1.9 (1.5-2.3) | <0.001 | 102 | 146 | 1.4 (1.1-1.9) | 0.006 | 113 | 154 | 1.5 (1.2-1.9) | 0.001 |
| Men | 173 | 332 | 1.0 (0.9-1.3) | NS | 80 | 101 | 1.6 (1.2-2.1) | 0.002 | 63 | 79 | 1.6 (1.2-2.2) | 0.006 | 84 | 119 | 1.4 (1.1-1.9) | 0.015 |
| Women | 123 | 231 | 1.1 (0.9-1.3) | NS | 82 | 73 | 2.3 (1.7-3.1) | <0.001 | 45 | 70 | 1.3 (0.9-1.9) | NS | 32 | 40 | 1.6 (1.0-2.6) | 0.045 |
| Indexyear1987-2000 | 107 | 199 | 1.1 (0.8-1.4) | NS | 73 | 78 | 1.9 (1.4-2.6) | <0.001 | 53 | 77 | 1.3 (1.0-2.0) | NS | 59 | 79 | 1.5 (1.1-2.1) | 0.019 |
| Indexyear 2001-2018 | 189 | 364 | 1.0 (0.9-1.2) | NS | 89 | 96 | 1.9 (1.4-2.5) | <0.001 | 55 | 72 | 1.5 (1.1-2.2) | 0.017 | 57 | 80 | 1.4 (1.0-2.0) | 0.040 |
| *Note:* NS: not statistically significant, ^a^ analysis only on patients with at least one known relative, ^b^ not enough cases with family history for cholesteatoma to run analysis | | | | | | | | | | | | | | | | |
